# Spontaneous Rupture of Multifocal Hepatic Artery Pseudoaneurysms in Systemic Lupus Erythematosus: A Rare Vasculopathic Emergency With Aberrant Hepatic Arterial Anatomy

**DOI:** 10.7759/cureus.114225

**Published:** 2026-08-09

**Authors:** Sourav Choudhury, Nicolae Serban Ioanid

**Affiliations:** 1 Hepato-Pancreato-Biliary and Transplant Surgery, St. Vincent's University Hospital, Dublin, IRL; 2 Transplant Surgery, St. Vincent's University Hospital, Dublin, IRL

**Keywords:** endovascular therapy, hemoperitoneum, hepatic artery pseudoaneurysm, systemic lupus erythematosus, visceral vasculopathy

## Abstract

Hepatic artery pseudoaneurysm is an uncommon but life-threatening vascular condition, and spontaneous rupture in a patient with systemic lupus erythematosus (SLE) is exceptionally rare. We report a 45-year-old woman with SLE who presented with acute abdominal distension, anaemia, and hemorrhagic shock. Computed tomographic mesenteric angiography demonstrated hemoperitoneum with multifocal hepatic artery pseudoaneurysms, including a ruptured pseudoaneurysm arising from a replaced right hepatic artery originating from the superior mesenteric artery, along with a second large left hepatic artery pseudoaneurysm. Emergency endovascular management was performed, achieving hemostasis while preserving hepatic perfusion. The patient recovered with supportive care and remained well at three-month follow-up. Early recognition of unexplained shock and abdominal symptoms in patients with connective tissue disease may facilitate timely imaging and life-saving intervention.

## Introduction

Hepatic artery pseudoaneurysm (HAP) is a rare but potentially fatal vascular lesion caused by disruption of the arterial wall, with a high risk of rupture and death if not recognized promptly [1]. Most cases occur after hepatobiliary intervention, trauma, pancreatitis, or liver transplantation; spontaneous HAP in the absence of recent instrumentation is distinctly uncommon (89.9% vs 10.1%) [1,2]. Inflammatory and autoimmune disorders, including systemic vasculitides and systemic lupus erythematosus (SLE), may predispose to the formation of visceral aneurysms or pseudoaneurysms through vessel wall inflammation and fragility. In these conditions, immune complex deposition and complement activation cause transmural necrotising vasculitis. This inflammatory injury weakens the arterial media and adventitia, predisposing to focal wall rupture that becomes contained by periarterial tissues, forming a pseudoaneurysm [3]. We describe a striking case of spontaneous rupture of multifocal HAP in a woman with SLE, further complicated by aberrant hepatic arterial anatomy, to highlight an unusual but important systemic vascular emergency.

## Case presentation

**Figure 1:**
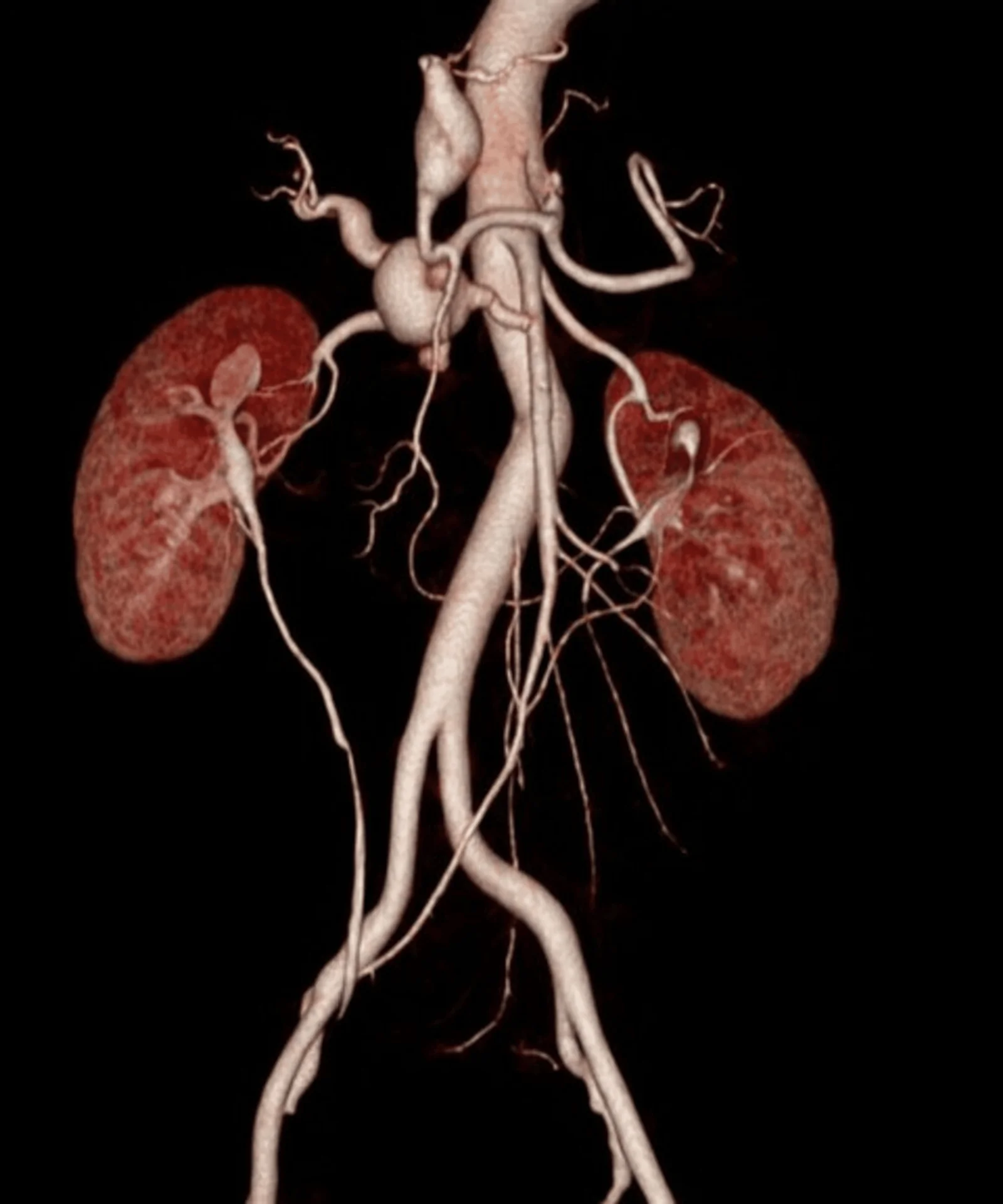
3D-rendered CT imaging of the aberrent right hepatic artery arising from the superior mesenteric artery with large pseudoaneurysm from both the hepatic arteries

A 45-year-old woman with known SLE for the last eight years presented to the emergency department with sudden abdominal distension and features of hypovolemic shock. She was drowsy and hypothermic. On physical examination, her abdomen was distended, tender but without any rigidity. There was no history of recent abdominal trauma, hepatobiliary intervention, or gastrointestinal bleeding. Initial evaluation showed profound anaemia with a haemoglobin level of 4 g/dL (normal range 11-14 g/dl) with a raised serum lactate level of 3.5 mmol/L (normal range <1 mmol/L). The patient was resuscitated with intravenous fluids and blood products, oxygen by mask and urinary catheterisation and showed haemodynamic improvement. She was further evaluated with computed tomographic mesenteric angiography, which demonstrated a large hemoperitoneum and multifocal HAPs. One pseudoaneurysm arose from a replaced right hepatic artery originating from the superior mesenteric artery, and another large pseudoaneurysm involved the left hepatic artery (Figure 1). Although active extravasation was not clearly demonstrated on all imaging phases, no alternate source of intra-abdominal bleeding was identified. In view of ongoing hemodynamic instability requiring vasopressor support, urgent endovascular treatment was undertaken.

**Figure 2:**
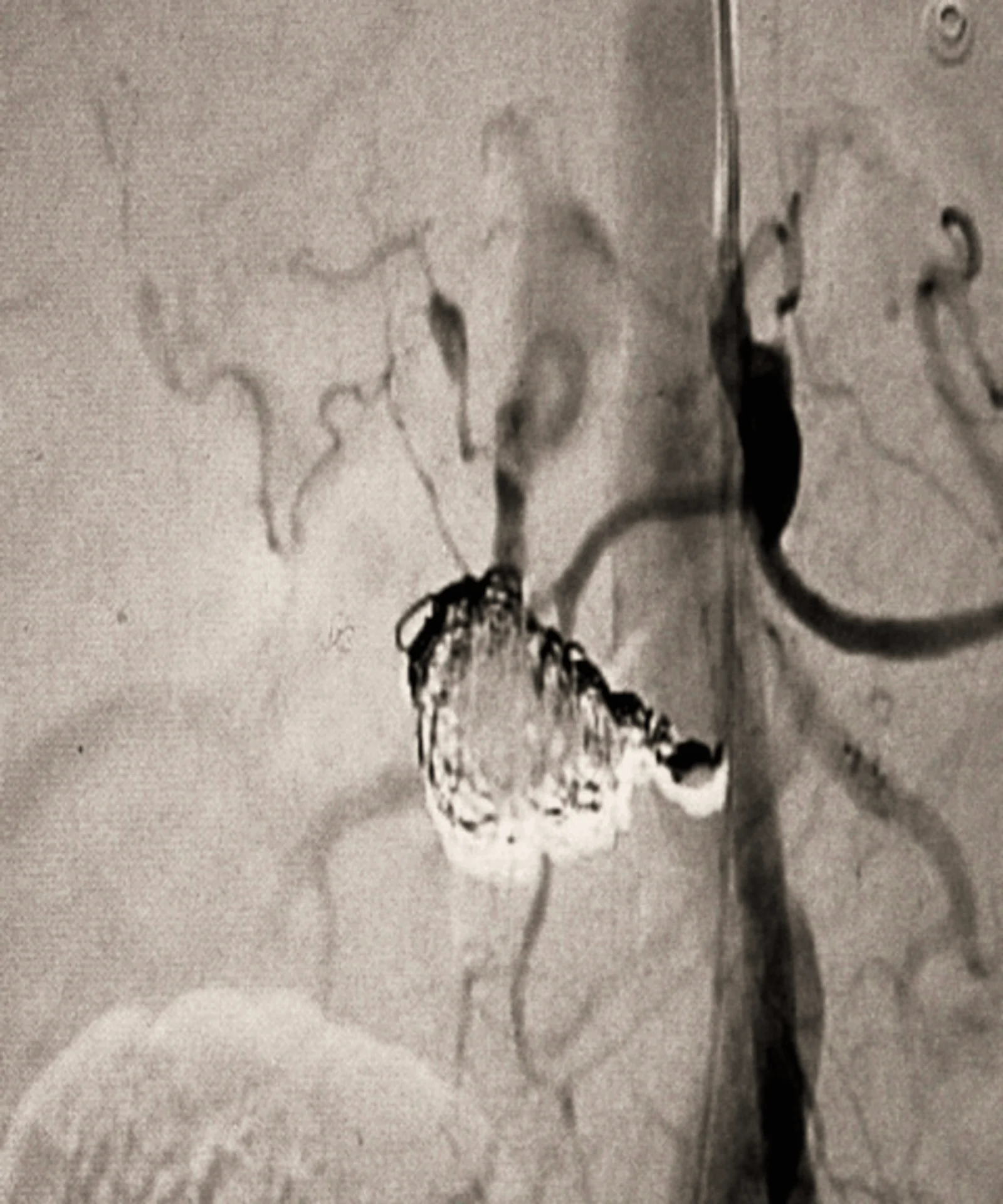
Direct angiogram and coiling of pseudoaneurysm of replaced right hepatic artery

Emergency angiography confirmed the complex vascular anatomy. Coil embolization of the right HAP was successfully performed (Figure 2). Management of the left HAP was more challenging because embolization risked significant hepatic ischemia. Therefore, a covered stent was deployed across the left hepatic artery lesion, successfully excluding the pseudoaneurysm while preserving arterial inflow to the liver.

**Figure 3:**
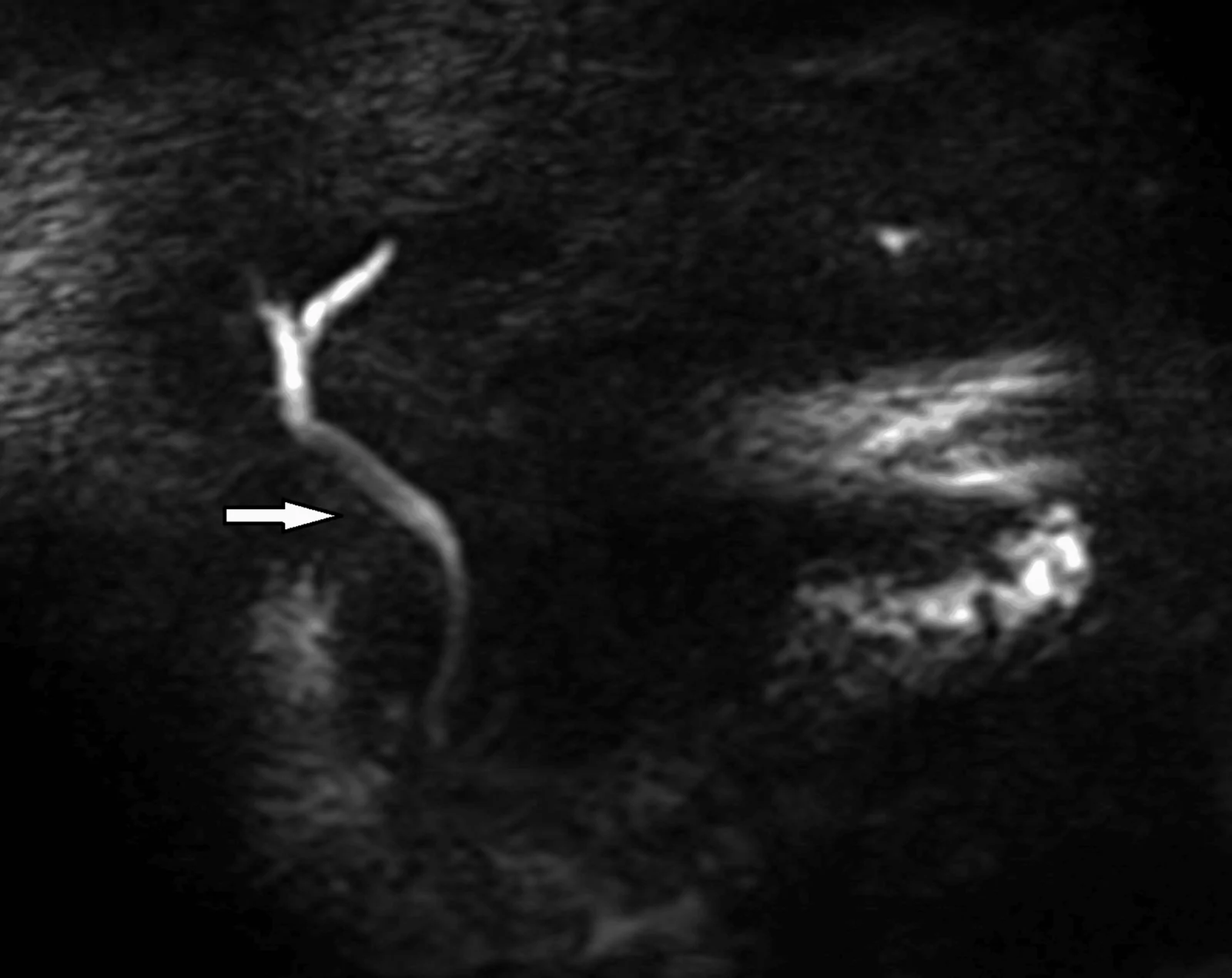
Magnetic resonance cholangiopancreatography picture showing the biliary tract (white arrow indicates common bile duct) without any biliopathy

**Figure 4:**
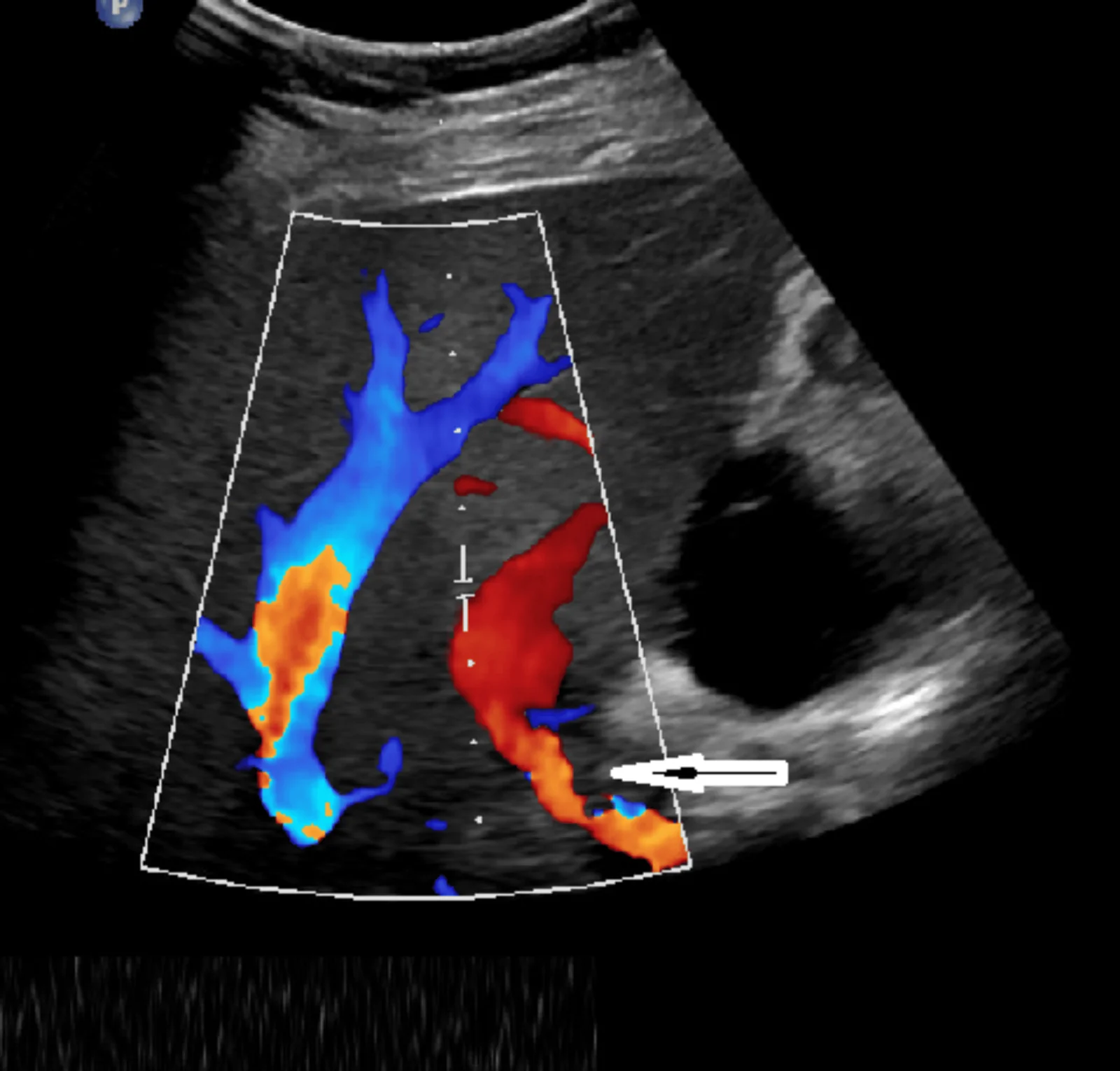
Colour doppler study after three months follow up showing good flow in the intrahepatic part of left hepatic artery (LHA) as indicated by the arrow.

The patient was monitored in the intensive care unit, and no further bleeding occurred. After the procedure, she developed a cholestatic pattern of liver enzyme derangement. Magnetic resonance cholangiopancreatography (Figure 3) showed no biliary obstruction or ischemic cholangiopathy, suggesting transient hepatic dysfunction related to the hemorrhagic insult and vascular intervention. Liver biochemistry gradually improved with conservative management. She was discharged after 12 days and remained clinically stable thereafter. At the three-month follow-up, Doppler ultrasonography (Figure 4) confirmed stent patency without recurrent bleeding. She was started on aspirin for stent maintenance and continued her routine medications, such as hydroxychloroquine and statins, which she was taking prior to her admission.

## Discussion

This case is notable for three reasons: spontaneous rupture, multifocal involvement, and occurrence in a patient with SLE. Although HAP is rare in any setting, spontaneous visceral pseudoaneurysm in connective tissue disease is particularly uncommon and may represent severe vasculopathy or vasculitis affecting medium-sized vessels. For dermatologists caring for patients with SLE, this case underscores that the disease spectrum extends beyond mucocutaneous findings and may rarely culminate in abrupt, life-threatening intra-abdominal haemorrhage.

The clinical presentation of HAP is often nonspecific. While some lesions are detected incidentally, rupture may present dramatically with abdominal pain, hemobilia, gastrointestinal bleeding, or hemorrhagic shock [1,4]. The classical Quincke triad of right upper quadrant pain, jaundice, and gastrointestinal bleeding is present in only a minority of cases [4]. Our patient presented instead with abdominal distension, profound anaemia, and hemoperitoneum, illustrating how diagnosis may be delayed if a vascular catastrophe is not considered early.

Prompt imaging is crucial. Computed tomographic angiography is usually the first-line investigation because it can rapidly detect hemoperitoneum, delineate vascular anatomy, and identify pseudoaneurysms or active extravasation. Digital subtraction angiography remains the diagnostic gold standard and simultaneously allows definitive therapy. In the present case, angiography was especially valuable because aberrant hepatic arterial anatomy complicated both interpretation and treatment planning.

Endovascular therapy is now the preferred treatment for HAP because it can achieve rapid haemorrhage control with lower morbidity than open surgery [2]. Choice of technique depends on anatomy, hemodynamic status, and the need to preserve downstream perfusion. Coil embolization is effective for emergency occlusion of the bleeding vessel, whereas covered stent placement is particularly useful when hepatic arterial flow must be maintained [4]. Our patient required both approaches: coil embolization for the ruptured right-sided lesion and stent-graft exclusion for the left HAP to reduce the risk of hepatic ischemia.

The aberrant hepatic arterial anatomy further complicated this case. A replaced right hepatic artery arising from the superior mesenteric artery is a recognised anatomical variant and can influence both the pattern of haemorrhage and the endovascular strategy. In patients with SLE, the threshold for considering an underlying vascular event should be low when unexplained abdominal pain, falling haemoglobin, or shock develops. Although there are no standardised recommendations for screening or prophylactic treatment of visceral pseudoaneurysms in SLE, this case highlights the importance of urgent multidisciplinary collaboration among emergency physicians, internists, dermatologists/rheumatologists, radiologists, and intensivists.

## Conclusions

Spontaneous rupture of a HAP is a rare but devastating vascular emergency that may occur in the setting of SLE-associated vasculopathy. In patients with connective tissue disease who present with sudden abdominal symptoms, severe anaemia, or shock, clinicians should promptly consider visceral vascular complications and obtain urgent angiographic imaging. Early endovascular intervention can be lifesaving, even in the presence of complex aberrant anatomy.
